# Efficacy of gastric decompression after pancreatic surgery: a systematic review and meta-analysis

**DOI:** 10.1186/s12876-020-01265-4

**Published:** 2020-04-25

**Authors:** Jia Gao, Xinchun Liu, Haoran Wang, Rongchao Ying

**Affiliations:** 1 Department of General Surgery, The Affiliated Hangzhou Hospital of Nanjing Medical University, Huansha Road 261, Hangzhou, 310000 Zhejiang Province China; 2 Department of General Surgery, Affiliated Hangzhou First People’s Hospital of Zhejiang University School of Medicine, Huansha Road 261, Hangzhou, 310000 Zhejiang Province China

**Keywords:** Pancreatic surgery, Gastric decompression, Complication, Meta-analysis

## Abstract

**Background:**

Gastric decompression after pancreatic surgery has been a routine procedure for many years. However, this procedure has often been waived in non-pancreatic abdominal surgeries. The aim of this meta-analysis was to determine the necessity of routine gastric decompression (RGD) following pancreatic surgery.

**Methods:**

PubMed, the Cochrane Library, EMBASE, and Web of Science were systematically searched to identify relevant studies comparing outcomes of RGD and no gastric decompression (NGD) after pancreatic surgery. The overall complications, major complications, mortality, delayed gastric emptying (DGE); clinically relevant DGE (CR-DGE), postoperative pancreatic fistula (POPF), clinically relevant POPF (CR-POPF), secondary gastric decompression, and the length of hospital stay were evaluated.

**Results:**

A total of six comparative studies with a total of 940 patients were included. There were no differences between RGD and NGD groups in terms of the overall complications (OR = 1.73, 95% CI: 0.60–5.00; *p* = 0.31), major complications (OR = 2.22, 95% CI: 1.00–4.91; *p* = 0.05), incidence of secondary gastric decompression (OR = 1.19, 95% CI: 0.60–2.02; *p* = 0.61), incidence of overall DGE (OR = 2.74; 95% CI: 0.88–8.56; *p* = 0.08; I^2^ = 88%), incidence of CR-POPF (OR = 1.28, 95% CI: 0.76–2.15; *p* = 0.36), and incidence of POPF (OR = 1.31, 95% CI: 0.81–2.14; *p* = 0.27). However, RGD was associated with a higher incidence of CR-DGE (OR = 5.45; 95% CI: 2.68–11.09; *p* < 0.001, I^2^ = 35%), a higher rate of mortality (OR = 1.53; 95% CI: 1.05–2.24; *p* = 0.03; I^2^ = 83%), and a longer length of hospital stay (WMD = 5.43, 95% CI: 0.30 to 10.56; *p* = 0.04; I^2^ = 93%).

**Conclusions:**

Routine gastric decompression in patients after pancreatic surgery was not associated with a better recovery, and may be unnecessary after pancreatic surgery.

## Background

Since its first introduction in the 1930s for the treatment of intestinal obstruction and postoperative ileus, routine gastric decompression (RGD) has long been considered the standard of care following elective abdominal procedures [1, 2]. RGD was believed to accelerate the recovery of gastrointestinal function, to prevent the risk of gastric stasis and resultant nausea, vomiting, and to reduce anastomotic leakage [3, 4]. However, the routine use of RGD after abdominal surgery has been increasingly questioned, especially with the introduction of Enhanced Recovery After Surgery (ERAS) [5]. Although evidence needs to be strengthened, the 2012 ERAS guidelines for perioperative care for pancreaticoduodenectomy (PD) strongly recommended avoiding the pre-emptive use of nasogastric tubes postoperatively as it does not improve outcomes [6].

Emerging evidences have demonstrated that it is safe to omit routine postoperative nasogastric decompression after esophagectomy [7], gastrectomy [8–10], liver [11], and colorectal surgery [12]. In contrast to hepatic and gastric surgery, a consensus about the impact of RGD after pancreatic resection has not yet been reached. This is partially due to the fact that the pancreas is a fragile organ, and pancreatic surgery is regarded as one of the most complicated operations in the abdominal area. Although the mortality rate after PD is now less than 5% in many centers, the morbidity some previous studies provide evidence that, and hemorrhage remains high [13, 14]. Most surgeons still routinely perform RGD after pancreatic resection in the hope that RGD would reduce postoperative complications and contribute to postoperative recovery. However, some studies have found that avoiding the use of a nasogastric tube actually speeds the return of bowel function, decreases pulmonary complications and is not associated with an increase in the anastomotic leak; thus, RGD after pancreatic resections may not be necessary for the majority of patients [15, 16].

Previous studies have been retrospective on small sample sizes, making it difficult hard to reach a convincing decision about whether RGD is beneficial after pancreatic resections. Therefore, we conducted this systematic review and meta-analysis and aimed to assess the necessity of RGD in patients after pancreatic resections.

## Methods

This study was performed according to the Preferred Reporting Items for Systematic Reviews and Meta-Analysis (PRISMA) statement [17]. After the establishment of the search strategy, two reviewers (J. G. and X. L.) independently performed the study selection, data extraction, study quality assessment, and critical appraisal. Disagreements were resolved by discussion with a third reviewer (R. Y.).

### Literature search

A systematic literature search was conducted in PubMed, EMBASE, Cochrane Central Register of Controlled Trials (CENTRAL), and Web of Science for eligible studies comparing RGD with no gastric decompression (NGD) after pancreatic resection. References of relevant articles were also reviewed to identify potentially eligible studies.

The search strategy was (“pancreaticoduodenectomy” OR “pancreatoduodenectomy” OR “Whipple” OR “pancreatic resection” OR “pancreatectomy” OR “pancreatic surgery”) AND (“nasogastric tube” OR “gastrostomy” OR “gastric decompression”). The search strategy was adapted to the databases accordingly. The last search was conducted on Nov 3, 2019.

### Study selection criteria

All types of original study articles that performed a comparison between RGD and NGD after pancreatic resections were considered. No restrictions were made regarding the methods of gastric decompression (nasogastric tube versus gastrostomy).

Publications were excluded if they meet any of the following criteria: (1) articles were published as case reports, conference abstracts, letters to the editor, or reviews; (2) articles were published in any language other than English; (3) articles compared routine gastric decompression to selective gastric decompression, rather than to no-gastric decompression.

### Outcome measures

The primary outcomes of this meta-analysis were the incidence of overall complications and major complications after pancreatic resections. Secondary outcomes included delayed gastric emptying (DGE), postoperative pancreatic fistula (POPF), and postoperative mortality. Secondary gastric decompression and the length of hospital stay were also analyzed.

### Data extraction

The extracted data included study characteristics (study design, study period, sample size, and investigated surgical procedure), patient characteristics (age, sex, body mass index), and outcome measures [POPF), DGE, hemorrhage, intra-abdominal fluid collection/abscess, bile leakage, wound infection, pneumonia, overall morbidity, mortality, reoperations, length of hospital stay].

For the outcomes of interest, when the continuous variable was reported only as medians and ranges, the methods of Hozo et al. [18] and Wan et al. [19] were applied to calculate means and standard deviations.

When data were reported in more than one article or were analyzed using two statistical methods to analyze patients from the same database, the one with larger data sets were included.

### Study quality assessment

The quality of included studies was assessed using the Newcastle–Ottawa Scale for cohort studies (NOS). Included studies were ranked with a maximum of 9 points, including three parts: “selection” (four elements), “comparability” (one element) and “outcome” (three elements). Cohort studies with an NOS score < 6 were considered of moderate or low quality.

### Statistical analysis

Statistical analyses were performed using Review Manager 5.3 for Windows. For continuous outcomes, weighted mean differences (MD) and corresponding 95% confidence intervals (CI) were calculated by the inverse variance method. For dichotomous outcomes, odds ratios (OR) and the corresponding 95% CI were calculated by the Mantel–Haenszel (MH) method. For the assessment of statistical heterogeneity, I^2^ statistics were performed. When I^2^ > 50%, statistical heterogeneity was considered high. Due to the clinical heterogeneity and for a relatively conservative perspective, a random-effects model was chosen for the meta-analyses regardless of the absence of statistical heterogeneity. Publication bias for the primary outcome was analyzed using funnel plots and Egger’s test. *P* < 0.05 was considered to be statistically significant, and the 95% CI was set for efficiency measures.

## Results

### Literature search

**Fig. 1:**
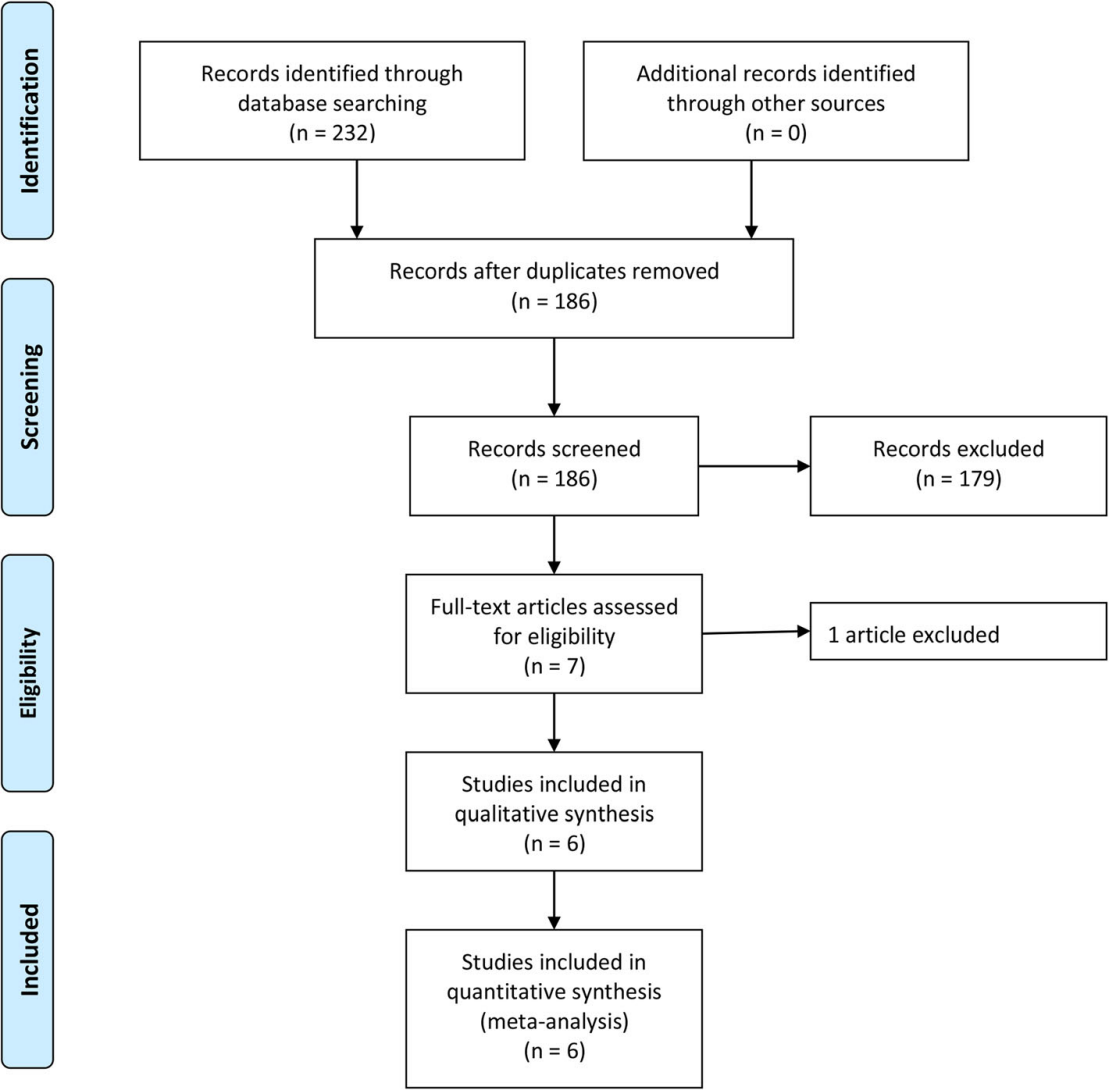
Flow chart

Figure 1 depicts the screening and selection process of the literature in accordance with PRISMA guidelines. Initially, a total of 232 studies were identified from the databases. After discarding duplicates an unrelated study according to the exclusion criteria, seven full-text articles were reviewed to assess further eligibility. In addition, one study was excluded because some patients in the NGD group underwent nasogastric tube insertion (NGT) [20]. Finally, six studies were included in the systematic review and meta-analysis [5, 15, 16, 21–23]. The final analysis included a total 940 patients, with 484 patients in the RGD group, and 456 patients in the NGD group.

### Study quality assessment

Study quality was assessed using the modification of the Newcastle-Ottawa scale for the cohort study. The median quality score of the studies according to the Newcastle-Ottawa Scale was 7.

### Baseline study characteristics

All six studies were non-randomized studies, four of which used history as the control. Two studies were conducted in the USA, two in Korea, one in France, and one in Norway. Sample sizes ranged from 41 to 231. The studies were published between 2011 and 2019, and the study period ranged from 1994 to 2016. The baseline characteristics of the six included studies are summarized in Table 1.

**Table 1:** The baseline characteristics of the six included studies

Reference	Publication Year	Country	Study design	Study period	Group	No. of Patients	Sex (M/F)	Type of procedure	Operation time (min)	Blood loss (mL)
Gaignard et al [15]	2018	France	Time cohort	2014–2015	NCT +	99	62:37	PD	270 (210, 337)	NA
					NCT -	40	25:15	PD	300 (249, 343)	NA
Park et al [16]	2016	Korea	Time cohort	2009–2014	Gastrostomy-	112	64:48	PPPD	474.8 ± 129.9	950 (140–2600)
					Gastrostomy+	116	52:64	PPPD	484.2 ± 115.0	775 (100–2700)
Roland et al [21]	2012	USA	Time cohort	1994–2011	NGT-	75	32:43	PD:56;	NA	NA
								DP: 14;		
								Others: 5		
					NGT+	156	66:90	PD: 113;	NA	NA
								DP:32;		
								Others:11		
Fisher et al [22]	2011	USA	Time cohort	2008–2010	NGT-	50	20:30	Whipple: 33	NA	250 (150–500)IQ
								DP: 17		
					NGT+	50	24:26	Whipple: 31	NA	400 (200–700)IQ
								DP: 19		
Choi et al [23]	2011	Korea	Retrospective	2001–2007	NGT-	23	14:9	Whipple: 16	528 ± 113	1178 ± 506
								PPPD: 6		
								Others: 1		
					NGT+	18	9:9	Whipple: 15	503 ± 88	922 ± 357
								PPPD: 1		
								Others: 2		
D. Kleive et al [5]	2019	Norway	Time cohort	2015–2016	NGT+	45	14:31	PD standard:16	372 ± 81.1	350 (50–3100)
								Pylorus preserving:29		
					NGT-	156	85:71	PD standard:47	347 ± 86.3	200 (50–3700)
								Pylorus preserving:109		

### Primary outcomes

#### Overall complications

**Fig. 2:**
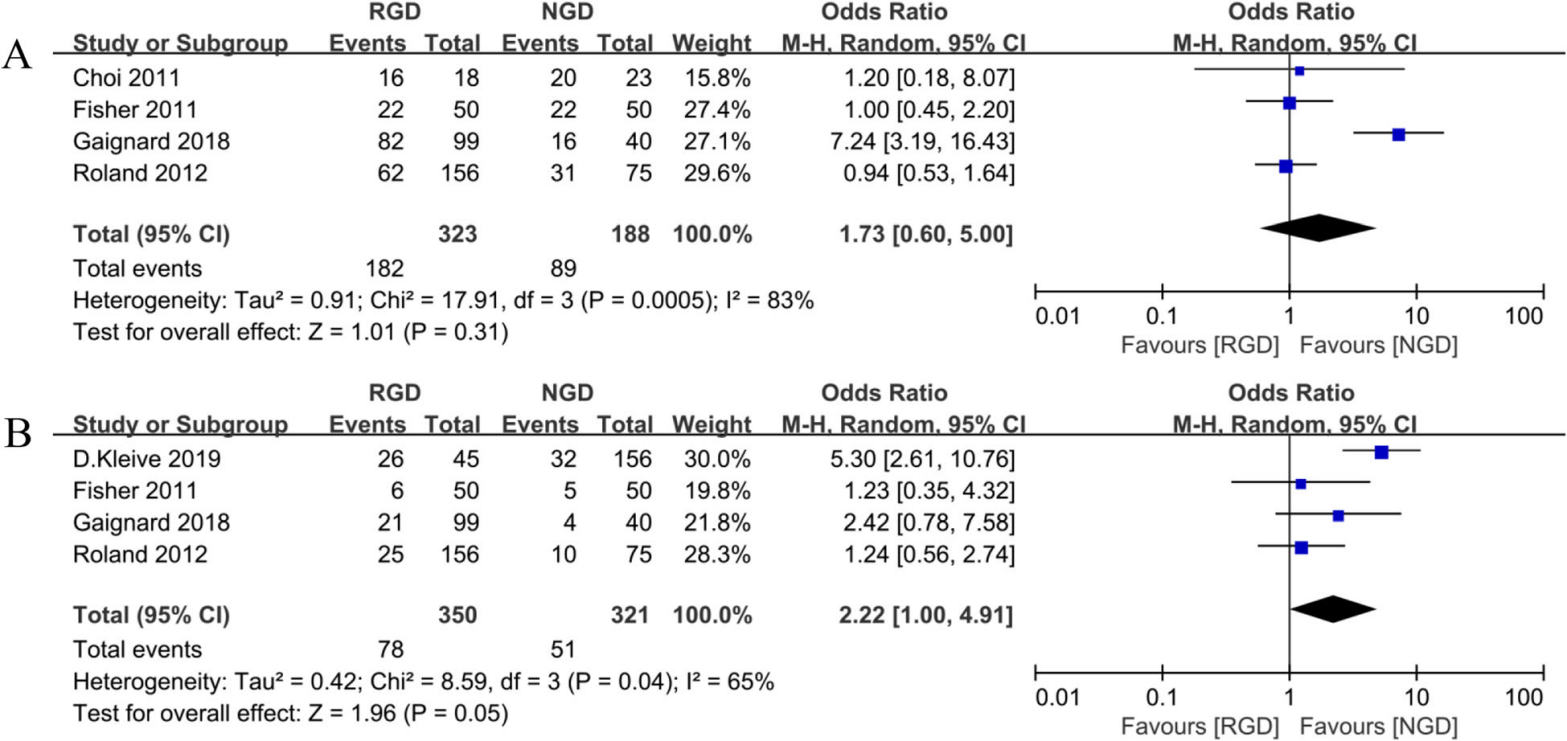
Meta-analysis comparing the primary outcomes (**a** overall complications; **b** major complications) between RGD and NGD groups

Five studies reported on postoperative complications [15, 16, 21–23]. Overall complications were defined and graded using the following: Common Terminology Criteria for Adverse Events CTCAE (v4.0) (Grade 1–5) [22]; the international Clavien-Dindo grading system [24]; the 5-grade scale described by DeOliveira et al. [24]. However, Park et al. excluded delayed gastric emptying, pancreatic fistula, and gastrostomy site infection from the complications rate [16]. Therefore, the remaining four studies were included for the meta-analysis. Moreover, there were no significant difference in overall complications between the two groups (OR = 1.73, 95% CI: 0.60–5.00; *p* = 0.31; Fig. 2a).

#### Major complications

Major complications were reported in four studies [5, 15, 21, 22] and were defined as: 1) accordion grade ≥ 3 complications [25]; 2) complications ≥ Grade III, which were graded on severity using the Common Terminology Criteria for Adverse Events CTCAE (v4.0) (Grade 1–5) [22]; 3) Dindo-Clavien grade ≥ 3a complications [24]; 4) complications ≥ III according to the 5-grade scale described by DeOliveira et al. [24]. Meta-analysis showed no significant difference between the two groups (OR = 2.22, 95% CI: 1.00–4.91; *p* = 0.05; Fig. 2b).

### Secondary outcomes

#### Secondary gastric decompression

**Fig. 3:**
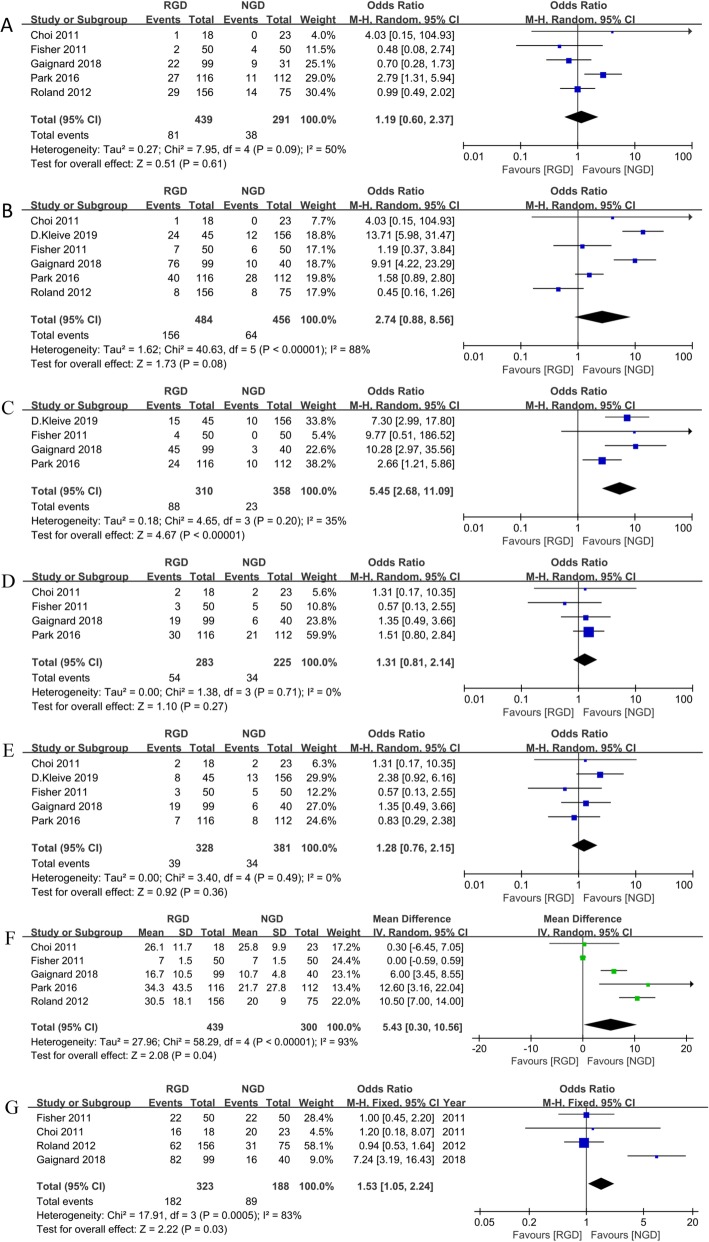
Meta-analysis comparing the secondary outcomes (**a** secondary gastric decompression; **b** DGE; **c** CR-DGE; **d** POPF; **e** CR-POPF; **f** postoperative hospital stay; **g** mortality) between RGD and NGD groups

Five studies reported the incidence of secondary gastric decompression in the RGD and decompression in the NGD group [15, 16, 21–23]. Meta-analysis revealed that there was no significant difference between the postoperative reinsertion rate in the decompression group and insertion rate in the non-decompression group (OR = 1.19, 95% CI: 0.60–2.37; *p* = 0.61; Fig. 3a).

#### DGE

All six studies reported results of DGE, four of which using the suggested definition of DGE by the International Study Group of Pancreatic Surgery (ISGPS) [5, 15, 16, 22, 26] while the remaining two studies do not. DGE were defined as gastric stasis requiring nasogastric intubation for >10 days or the inability to tolerate a regular diet on the 14th post-operative day [23], and "nausea and vomiting requiring NGT reinsertion for longer than 7 days combined with the inability to take oral nutrition or hydration by postoperative day 10 or the inability to tolerate oral intake, prolonging the patient’s hospital stay by more than 2 days" [21] in the remaining two studies, respectively. These studies investigated the incidence of DGE, and no significant difference was found between the two groups (OR = 2.74; 95% CI: 0.88–8.56; *p* = 0.08; I^2^ = 88%; Fig. 3b).

#### CR-DGE

Four studies reported results of CR-DGE [5, 15, 16, 22] and showed a significant difference in terms of CR-DGE between the two groups (OR = 5.45; 95% CI: 2.68–11.09; *p* < 0.001, I^2^ = 35%; Fig. 3c), favoring the NGD group. CR-DGE were defined as grade ≥ B DGE according to ISGPS [26].

#### POPF

Five studies reported clinically relevant POPF (CR-POPF) rates [5, 15, 16, 22, 23], and four reported POPF rate [15, 16, 22, 23]. POPF were defined as follows: 1) persistent secretions of bilirubin-rich drainage fluid >50 mL per day or after the 10th post-operative day; 2) the three-tiered definition proposed by the International Study Group on Pancreatic Fistula (ISGPF) [27]; 3) the 2016 definition of ISGPF [28]; and 4) "output via an operative drain of any measurable volume of drain fluid on or after postoperative day 3 with an amylase greater than three times the upper normal serum level ([300 IU/L) according to ISGPF definition" [29]. CR-POPF were defined as grade ≥ B POPF according to ISGPF. There was no difference between the two group in terms of POPF (OR = 1.31; 95% CI: 0.81–2.14; *p* = 0.27, I^2^ = 0%; Fig. 3d) and CR-POPF (OR = 1.28; 95% CI: 0.76–2.15; *p* = 0.36, I^2^ = 0%; Fig. 3e).

#### Postoperative hospital stay

Only one study reported the mean length of hospital stay with precise standard deviations [23]; the other studies reported median values with corresponding ranges or interquartile ranges [15, 16, 21, 22]. The meta-analysis identified high heterogeneity (I^2^ = 93%). However, there was a significant difference between the patients treated without RGD and those with RGD, favouring NGD (WMD = 5.43, 95% CI: 0.30 to 10.56; *p* = 0.04; I^2^ = 93%; Fig. 3f).

#### Mortality

Four studies reported mortality, and there was a significant difference between the two groups (OR = 1.53; 95% CI: 1.05–2.24; *p* = 0.03, I^2^ = 83%; Fig. 3g) [15, 21–23], favoring NGD.

## Discussion

### Main findings

This meta-analysis has shown that there was no difference in terms of overall complications, major complications, incidence of secondary gastric decompression, incidence of overall DGE, incidence of CR-POPF and incidence of POPF between RGD group and NGD group following pancreatic surgery. RGD was associated with a higher incidence of CR-DGE, a higher rate of mortality, and a longer length of hospital stay.

### Comparison with previous studies

These results are similar to those of previous meta-analyses, which showed that nasogastric decompression brings no benefit in non-pancreatic abdominal surgery, such as esophagectomy [7], gastrectomy [8, 10], or colorectal resection [12, 30]. In esophagectomy, in a systematic analysis of 608 patients, Weijs et al. showed no significant difference in adverse outcomes between nasogastric decompression or no nasogastric decompression following esophagectomy [7]. In gastrectomy for gastric cancer, Yang et al., with a meta-analysis of 717 patients from five RCTs, found that time to oral diet was significantly shortened in the no-decompression group, while time to flatus, anastomotic leakage, pulmonary complications, length of hospital stay, morbidity, and mortality were similar in both groups [8]. The finding was further confirmed by Wei et al. [10]. In a meta-analysis of 1141 patients, which found that nasogastric or nasojejunal decompression neither facilitated the recovery of bowel function nor reduced the risk of postoperative complications after gastrectomy for gastric cancer. Although the absence of routine placement of RGD has been clearly proved in other digestive surgeries and is now recommended after pancreatic surgery (including PD) by ERAS [31], routine nasogastric tube decompression is still practiced by many surgeons treating pancreatic cancer. This phenomenon can be attributed to several reasons. First, previous studies on the necessity of RGD after pancreatic resections were single-institution, retrospective studies with relatively small sample sizes. Therefore, the ERAS recommendation is based only on moderate evidence. Second, the high morbidity after pancreatic resection contributes to this practice. DGE is one of the most common complications after pancreatic surgery, especially following PD, which negatively impacts the quality of life, prolongs the hospital stay, and increases hospital costs. Although its pathophysiology remains unclear, it has discouraged many surgeons from abandoning this practice. Routine nasogastric tube placement after abdominal surgery is thought to prevent postoperative nausea and vomiting and abdominal distention by gastric decompression; these are the core symptoms of DGE. Third, because NG tube has been used following gastrointestinal anastomoses for several decades, it is difficult to change the clinical habit and radically stop using routine gastric decompression [32, 33].

Instead of absolutely prohibiting RGD after pancreatic surgery, some surgeons preferred a more conservative method, namely selective NGT usage, such as when they unable to extubate the patient postoperatively [20]. In their retrospective study with 250 patients, Kunstman et al. found that patients in the selective use of RGD had decreased incidence of delayed gastric emptying, length of stay, and time to dietary tolerance [20]. Nevertheless, the authors agreed that RGD could be omitted in many cases.

Previous studies in non-pancreatic surgery have found that pulmonary complications, such as atelectasis and pneumonia, occur more frequently in patients with a nasogastric tube than in those without. These findings were also confirmed in pancreatic resections; however, because only two studies reported this complication, a meta-analysis was not done in this study.

### Limitations

The results of this meta-analysis should be interpreted with caution due to several reasons. First, all six included studies employed a non-randomized design, which carries the potential for selection bias. However, four of the studies used historical controls, which may mitigate the selection bias. Second, there was heterogeneity between the two groups in terms of surgical procedures, histological grades, as well as tumor stage. Third, secondary outcomes were not reported by all the studies. Therefore, many important outcomes, such as pulmonary complications and time to dietary tolerance, were not analyzed, or only a limited number of patients were included for the meta-analysis of secondary outcomes, which might affect the reliability of the results. Finally, some studies did not directly provide means and SDs, and the Hozo algorithm was adopted to estimate means and SDs based on median and range, which may have introduced bias.

## Conclusions

Based on the available evidence, RGD is not associated with better postoperative outcomes after pancreatic surgery. Therefore, RGD after pancreatic surgery seems unnecessary. Further well-designed randomized controlled trials are needed to confirm this finding.

## Data Availability

All data generated or analysed during this study are included in this published article.

## Abbreviations

RGD: Routine gastric decompression
ERAS: Enhanced Recovery After Surgery
PD: Pancreaticoduodenectomy
PRISMA: Preferred Reporting Items for Systematic Reviews and Meta-Analysis
NGD: No gastric decompression
DGE: Delayed gastric emptying
POPF: Postoperative pancreatic fistula
NOS: Newcastle–Ottawa Scale
MD: Weighted mean differences
CI: Confidence intervals
OR: Odds ratios
MH: Mantel–Haenszel
CR-DGE: Clinically-relevant DGE
CR-POPF: Clinically-relevant POPF
ISGPF: International Study Group on Pancreatic Fistula
NGT: Nasogastric tube insertion

## References

[1] Wangensteen OH, Paine JR (1933). Treatment of acute intestinal obstruction by suction with the duodenal tube. J Am Med Assoc.

[2] Sagar PM, Kruegener G, MacFie J (1992). Nasogastric intubation and elective abdominal surgery. Br J Surg.

[3] Kingma BF, Steenhagen E, Ruurda JP, van Hillegersberg R (2017). Nutritional aspects of enhanced recovery after esophagectomy with gastric conduit reconstruction. J Surg Oncol.

[4] Nelson R, Tse B, Edwards S (2005). Systematic review of prophylactic nasogastric decompression after abdominal operations. Br J Surg.

[5] Kleive D, Sahakyan MA, Labori KJ, Lassen K (2019). Nasogastric tube on demand is rarely necessary after Pancreatoduodenectomy within an enhanced recovery pathway. World J Surg.

[6] Lassen K, Coolsen MM, Slim K, Carli F, de Aguilar-Nascimento JE, Schafer M, Parks RW, Fearon KC, Lobo DN, Demartines N (2012). Guidelines for perioperative care for pancreaticoduodenectomy: enhanced recovery after surgery (ERAS(R)) society recommendations. Clin Nutr.

[7] Weijs TJ, Kumagai K, Berkelmans GH, Nieuwenhuijzen GA, Nilsson M, Luyer MD (2017). Nasogastric decompression following esophagectomy: a systematic literature review and meta-analysis. Dis Esophagus.

[8] Yang Z, Zheng Q, Wang Z (2008). Meta-analysis of the need for nasogastric or nasojejunal decompression after gastrectomy for gastric cancer. Br J Surg.

[9] Wang D, Li T, Yu J, Hu Y, Liu H, Li G (2015). Is nasogastric or nasojejunal decompression necessary following gastrectomy for gastric cancer? A systematic review and meta-analysis of randomised controlled trials. J Gastrointest Surg.

[10] Wei ZW, Li JL, Li ZS, Hao YT, He YL, Chen W, Zhang CH (2014). Systematic review of nasogastric or nasojejunal decompression after gastrectomy for gastric cancer. Eur J Surg Oncol.

[11] Pessaux P, Regimbeau JM, Dondero F, Plasse M, Mantz J, Belghiti J (2007). Randomized clinical trial evaluating the need for routine nasogastric decompression after elective hepatic resection. Br J Surg.

[12] Rao W, Zhang X, Zhang J, Yan R, Hu Z, Wang Q (2011). The role of nasogastric tube in decompression after elective colon and rectum surgery: a meta-analysis. Int J Color Dis.

[13] Yoshioka R, Yasunaga H, Hasegawa K, Horiguchi H, Fushimi K, Aoki T, Sakamoto Y, Sugawara Y, Kokudo N (2014). Impact of hospital volume on hospital mortality, length of stay and total costs after pancreaticoduodenectomy. Br J Surg.

[14] Denbo JW, Bruno M, Dewhurst W, Kim MP, Tzeng CW, Aloia TA, Soliz J, Speer BB, Lee JE, Katz MHG. Risk-stratified clinical pathways decrease the duration of hospitalization and costs of perioperative care after pancreatectomy. Surgery. 2018. 10.1016/j.surg.2018.04.014 PMC7265789 29807648

[15] Gaignard E, Bergeat D, Courtin-Tanguy L, Rayar M, Merdrignac A, Robin F, Boudjema K, Beloeil H, Meunier B, Sulpice L. Is systematic nasogastric decompression after pancreaticoduodenectomy really necessary? Langenbecks Arch Surg. 2018. 10.1007/s00423-018-1688-8 29943225

[16] Park JS, Kim JY, Kim JK, Yoon DS (2016). Should gastric decompression be a routine procedure in patients who undergo pylorus-preserving Pancreatoduodenectomy?. World J Surg.

[17] Moher D, Liberati A, Tetzlaff J, Altman DG, Group P (2009). Preferred reporting items for systematic reviews and meta-analyses: the PRISMA statement. Ann Intern Med.

[18] Hozo SP, Djulbegovic B, Hozo I (2005). Estimating the mean and variance from the median, range, and the size of a sample. BMC Med Res Methodol.

[19] Wan X, Wang W, Liu J, Tong T (2014). Estimating the sample mean and standard deviation from the sample size, median, range and/or interquartile range. BMC Med Res Methodol.

[20] Kunstman JW, Klemen ND, Fonseca AL, Araya DL, Salem RR (2013). Nasogastric drainage may be unnecessary after pancreaticoduodenectomy: a comparison of routine vs selective decompression. J Am Coll Surg.

[21] Roland CL, Mansour JC, Schwarz RE (2012). Routine nasogastric decompression is unnecessary after pancreatic resections. Arch Surg.

[22] Fisher WE, Hodges SE, Cruz G, Artinyan A, Silberfein EJ, Ahern CH, Jo E, Brunicardi FC (2011). Routine nasogastric suction may be unnecessary after a pancreatic resection. HPB (Oxford).

[23] Choi YY, Kim J, Seo D, Choi D, Kim MJ, Kim JH, Lee KJ, Hur KY (2011). Is routine nasogastric tube insertion necessary in pancreaticoduodenectomy?. J Korean Surg Soc.

[24] Dindo D, Demartines N, Clavien PA (2004). Classification of surgical complications: a new proposal with evaluation in a cohort of 6336 patients and results of a survey. Ann Surg.

[25] Strasberg SM, Linehan DC, Hawkins WG (2009). The accordion severity grading system of surgical complications. Ann Surg.

[26] Wente MN, Bassi C, Dervenis C, Fingerhut A, Gouma DJ, Izbicki JR, Neoptolemos JP, Padbury RT, Sarr MG, Traverso LW (2007). Delayed gastric emptying (DGE) after pancreatic surgery: a suggested definition by the international study Group of Pancreatic Surgery (ISGPS). Surgery.

[27] Butturini G, Daskalaki D, Molinari E, Scopelliti F, Casarotto A, Bassi C (2008). Pancreatic fistula: definition and current problems. J Hepato-Biliary-Pancreat Surg.

[28] Bassi C, Marchegiani G, Dervenis C, Sarr M, Abu Hilal M, Adham M, Allen P, Andersson R, Asbun HJ, Besselink MG (2017). The 2016 update of the international study group (ISGPS) definition and grading of postoperative pancreatic fistula: 11 years after. Surgery.

[29] Pulvirenti A, Ramera M, Bassi C (2017). Modifications in the International Study Group for Pancreatic Surgery (ISGPS) definition of postoperative pancreatic fistula. Transl Gastroenterol Hepatol.

[30] Bauer VP (2013). The evidence against prophylactic nasogastric intubation and Oral restriction. Clin Colon Rectal Surg.

[31] Klaiber U, Probst P, Strobel O, Michalski CW, Dorr-Harim C, Diener MK, Buchler MW, Hackert T (2018). Meta-analysis of delayed gastric emptying after pylorus-preserving versus pylorus-resecting pancreatoduodenectomy. Br J Surg.

[32] Park JS, Kim JY, Kim JK, Yoon DS (2017). Should gastric decompression be a routine procedure in patients who undergo pylorus-preserving Pancreatoduodenectomy?: reply. World J Surg.

[33] Tez M (2017). Who does benefit from nasogastric decompression? Patient or Surgeon. World J Surg.

